# Epigenomics and transcriptomics association study of blood pressure and incident diagnosis of hypertension in twins

**DOI:** 10.1038/s41440-025-02164-5

**Published:** 2025-02-19

**Authors:** Asmus Cosmos Skovgaard, Afsaneh M. Nejad, Hans Christian Beck, Qihua Tan, Mette Soerensen

**Affiliations:** 1 The Danish Twin Registry and the Research Unit for Epidemiology, Biostatistics and Biodemography, Department of Public Health, University of Southern Denmark, Odense, Denmark; 2 Department of Mathematics and Computer Science, University of Southern Denmark, Odense, Denmark; 3 Centre for Clinical Proteomics, Department of Clinical Biochemistry, Odense University Hospital, Odense, Denmark; 4 Department of Clinical Genetics, Odense University Hospital, Odense, Denmark

**Keywords:** Multi-Omics Association Study, Twins, Hypertension, Immune system, KMT2A gene

## Abstract

Hypertension is the most frequent health-related condition worldwide and is a primary risk factor for renal and cardiovascular diseases. However, the underlying molecular mechanisms are still poorly understood. To uncover these mechanisms, multi-omics studies have significant potential, but such studies are challenged by genetic and environmental confounding – an issue that can be effectively reduced by studying intra-pair differences in twins. Here, we coupled data on hypertension diagnoses from the nationwide Danish Patient Registry to a study population of 740 twins for whom genome-wide DNA methylation and gene expression data were available together with measurements of systolic and diastolic blood pressure. We investigated five phenotypes: incident hypertension cases, systolic blood pressure, diastolic blood pressure, hypertension (140/90 mmHg), and hypertension (130/80 mmHg). Statistical analyses were performed using Cox (incident cases) or linear (remaining) regression analyses at both the individual-level and twin pair-level. Significant genes (*p* < 0.05) at both levels and in both types of biological data were investigated by bioinformatic analyses, including gene set enrichment analysis and interaction network analysis. Overall, most of the identified pathways related to the immune system, particularly inflammation, and biology of vascular smooth muscle cell. Of specific genes, lysine methyltransferase 2 A (*KMT2A*) was found to be central for incident hypertension, ataxia-telangiectasia mutated (*ATM*) for systolic blood pressure, and beta-actin (*ACTB*) for diastolic blood pressure. Noteworthy, lysine methyltransferase 2A (*KMT2A*) was also identified in the systolic and diastolic blood pressure analyses. Here, we present novel biomarkers for hypertension. This study design is surprisingly rare in the field of hypertension.

**Figure:**
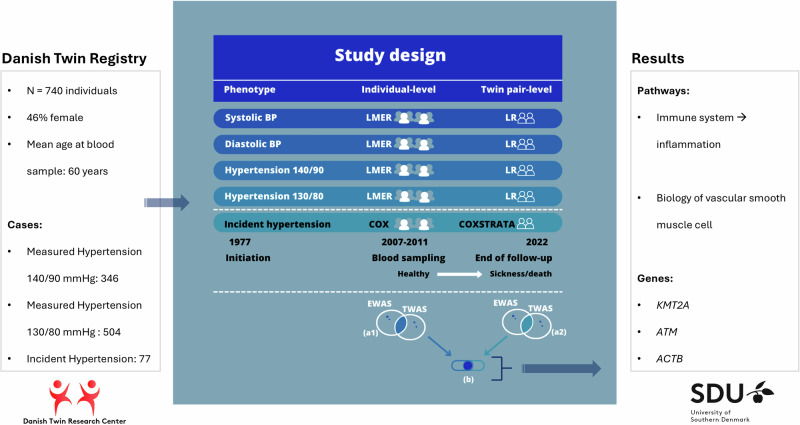
We identified biological pathways related to vascular smooth muscle cells and the immune system, particular inflammation, to be associated with hypertension and blood pressure. Of specific genes, we identified *KMT2A* (lysine methyltransferase 2A) to be central for blood pressure and hypertension development. ACTB beta-actin, ATM ataxiatelangiectasia mutated, BP blood pressure, EWAS epigenome-wide association studies, KMT2A lysine methyltransferase 2A, LMER linear mixed effect regression, LR linear regression, TWAS transcriptome-wide association studies.

We identified biological pathways related to vascular smooth muscle cells and the immune system, particular inflammation, to be associated with hypertension and blood pressure. Of specific genes, we identified *KMT2A* (lysine methyltransferase 2A) to be central for blood pressure and hypertension development. ACTB beta-actin, ATM ataxiatelangiectasia mutated, BP blood pressure, EWAS epigenome-wide association studies, KMT2A lysine methyltransferase 2A, LMER linear mixed effect regression, LR linear regression, TWAS transcriptome-wide association studies.

## Abstract (web summary)

We identified biological pathways related to vascular smooth muscle cells and the immune system, particular inflammation, to be associated with hypertension and blood pressure. Of specific genes, we identified KMT2A (lysine methyltransferase 2A) to be central for blood pressure and hypertension development. Abbreviations: ACTB: beta-actin, ATM: ataxia-telangiectasia mutated, BP: blood pressure, EWAS: epigenome-wide association studies, KMT2A: lysine methyltransferase 2A, LMER: linear mixed effect regression, LR: linear regression, TWAS: transcriptome-wide association studies.

## Introduction

High blood pressure (BP), also called hypertension, is the most frequent health-related condition worldwide and is estimated to affect 1.5 billion people globally [1, 2]. Moreover, hypertension causes 8.5 million deaths yearly as it is a primary risk factor for renal and cardiovascular diseases (CVD) [3]. For years, hypertension was defined as a systolic BP ≥ 140 mmHg and a diastolic BP ≥ 90 mmHg but was redefined to systolic BP/ diastolic BP ≥ 130/80 mmHg in 2018 [4]. Hypertension is a heterogenous condition and is regulated by genetic and epigenetic mechanisms that are poorly understood and can be affected by risk factors, such as aging, obesity, and genetic family history [4].

Currently, hypertension is treated with one or many classes of antihypertensive drugs irrespective of the pathophysiologic mechanisms leading to its genesis. Despite antihypertensive treatment, up to 50% of patients achieve blood pressure goals in clinical practice. Therefore, the discovery of new biomarkers for hypertension has several advantages, such as risk prediction and stratification, monitoring of the effect of treatment, in all leading to more individualized therapy and prevention of hypertensive end organ damage.

In the last decade, genome-wide association studies (GWAS) have identified over 1,000 genetic loci associated with hypertension, explaining about 6% of the heritability [5] and in which inflammation appears to be essential, as reviewed in [6]. However, most of the identified loci are in non-coding regions, for which the functional role is difficult to elucidate [7]. Moreover, GWAS provide little or no molecular evidence of gene causality. To overcome these limitations, genetic data have been integrated with high-throughput molecular data designed to investigate the epigenome, transcriptome, proteome, or metabolome, which potentially can reveal causal genes and essential molecular mechanisms involved in hypertension progression [8].

Increasing evidence suggests that epigenetic mechanisms underlie several complex diseases [9] in which the best studied epigenetic mechanism is DNA methylation at 5‘-cytosine-phosphate-guanine-3’ (CpG) [10], which is known to affect gene expression levels. Moreover, transcriptomics, also known as gene expression profiling, refers to the study of gene expression itself, as measured by RNA levels, and it has also been widely studied in relation to CVD [11]. Indeed, several epigenome-wide association studies (EWAS) have identified CpGs with differential methylation patterns for hypertension [6, 12–14], while several transcriptome-wide association studies (TWAS) have identified multiple genes associated with hypertension [15–18]. Many of the identified genes relate to inflammation processes, however, there is also some heterogeneity in the findings [6, 12–18]. One major challenge in these studies is that the association between non-genetic biomarkers and hypertension is confounded by genetic variation. Therefore, several twin studies have been conducted with the hope of resulting in less biased findings, as they can exclude genetic confounding. In such studies, twin pairs which are discordant for a given phenotype are investigated, e.g., one twin develops hypertension, while the co-twin does not [19]. This design is known as the discordant twin pair design and it is a statistically efficient approach, which has been estimated to reduce the requisite sample size to only one-tenth for a trait with a heritability of 60% for DNA methylation array data compared to the classical case-control design [20]. The heritability of hypertension has been reported to be 30–50% [8].

In 1072 Chinese twins, Hong X. et al. (2023) validated 16 and 20 CpG sites found in previous EWAS to be associated with systolic BP and diastolic BP, respectively. Several of these CpG sites were related to inflammation processes. Causality was also inspected, where they found that DNA methylation appeared to play an essential role in the development and progression of hypertension over time [14]. Regarding gene expression data, Yisong et al. (2018) validated 12 genes previously found in TWAS of hypertension by investigating 391 Finnish twins [17], while Lu-jie et al. (2022) identified five genes to be related to inflammation [18], which was supported by a meta-analysis of hypertension TWAS [15]. Hence, these twin studies confirm a role of inflammation in hypertension and point to the relevance of studying these types of biological variation in relation to hypertension.

As DNA methylation plays a key role in regulating gene expression, the integration of both types of data in relation to hypertension in twins provides the possibility to examine the complexity of hypertension and can potentially bring new important knowledge regarding the biological basis of hypertension and reveal new biomarkers and future hypertension therapeutic targets [8]. Yet, to the best of our knowledge, no such association study of hypertension has been conducted. However, one prediction model of blood pressure values was recently conducted, including DNA methylation and gene expression data [21], where several of the genes of the prediction model were related to immune system.

Hence, the purpose of this study was to integrate DNA methylation and gene expression data to reveal molecular mechanisms associated with BP and hypertension in twins using the discordant twin pair design.

## Materials and methods

**Fig. 1:**
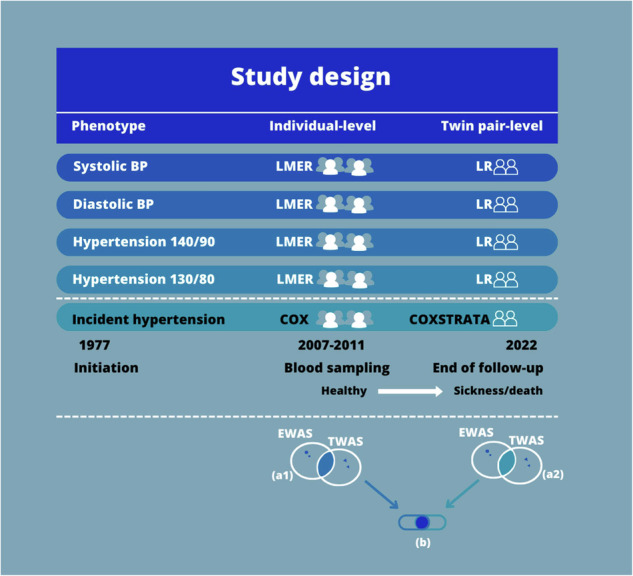
Study Design. We conducted gene set enrichment analyses (GSEA) with the aim to explore the biological pathways related to the five phenotypes in which the identified CpGs and probes take part. Before GSEA, the CpGs found in the EWAS with an FDR < 0.05, and the probes found in the TWAS with an FDR < 0.05 were identified in each statistical analysis. Next, for each of the statistical analyses, these CpG sites and probes were annotated to their gene names. As some different CpGs and probes might be annotated to the same gene, and as some CpGs might be annotated to multiple genes or different aliases, the unique genes were identified for the result of each statistical analysis. This resulted in 20 lists of gene names, i.e., five phenotypes times two analyses (individual-level and twin pair-level) times two kinds of molecular data. Afterwards, for each phenotype, the overlap in gene names of CpGs (EWAS) with FDR < 0.05 and the gene names of the probes (TWAS) with FDR < 0.05 were explored within each statistical analysis, for example the overlap in gene names between EWAS and TWAS in the incident hypertension Cox regression analysis at the individual-level corresponding to 1a in Fig. 1. This resulted in ten lists of EWAS-TWAS overlapping gene names (five phenotypes times two statistical models (an individual-level analysis and a twin pair-level analysis)). Next, for these ten lists, the overlap in EWAS-TWAS overlapping gene names from the individual-level analysis and, and EWAS-TWAS overlapping gene names from the twin pair analysis was found for each phenotype, corresponding to b in Fig. 1. This resulted in five overlaps of gene names identified in the individual-level analysis and confirmed in the twin pair level analysis, i.e., the genes confirmed when reducing the potential confounding due to shared genetic factors and early-life environment. Such genes must be considered the most relevant genes when exploring the association of molecular markers to hypertension. These five overlaps of confirmed genes (i.e. five phenotypes) were used for GSEA in the GSEA database with application of the Kyoto Encyclopedia of Genes and Genomes [41]. Moreover, genes present in some, or all, of the five overlaps of confirmed genes were explored. Finally, Interaction Networks were also conducted for the five overlaps of confirmed genes by application of StringApp in Cytoscape [42]. These steps were similarly done using a cut off *p* value < 0.05 instead of an FDR < 0.05 to explore the overlapping genes with *p* value < 0.05. Notes: BP is measured in mmHg. BP blood pressure, EWAS epigenome-wide association studies, LMER linear mixed effect regression, LR linear regression, TWAS transcriptome-wide association studies

Genome-wide epigenetic and genome-wide gene transcription data were available for 751 twins. From the same individuals, systolic BP and diastolic BP had been measured, and diagnoses for hypertension were obtained from the Danish National Patient Registry (DNPR) [22]. Eleven individuals did not have complete survey data; hence, 740 individuals (including 361 complete twin pairs) were left for analysis. The BP measurements and diagnoses, together with the two types of genome-wide data, were analyzed in the current study similarly to [23]. The study design is visualized in Fig. 1.

### Study population

The study population comprised 740 twins drawn from two population-based and nation-wide surveys from the Danish Twin Registry [24]: The Middle Age Danish Twin study (MADT) [24] and the Birthweight-Discordant Study (BD) [25]. The cohorts are further described in [23]. Of the included twins, the two types of genome-wide data were available in 476 twins from MADT 2008–2010 wave, and 275 twins from BD. Characteristics for the study population are summarized in Table 1. Data was collected through comprehensive interview-based questionaries and examinations, and blood sampling. Informed consent to participate in the cohorts was obtained from all participants, and the survey was approved by the Regional Scientific Ethical Committees for Southern Denmark (S-VF-19980072 and S-20090033) and conducted in accordance with the Helsinki II declaration.

**Table 1:** Characteristics of the participants used in the study

Number of participants	740
Women (%)	344 (46%)
Mean age (SD) (range), years	60 (13) (30–80)
Year of blood sample	2008-2011
Zygosity (MZ (%))	740 (100.0)
Smoker status (Never (%), Current (%), Former (%))	334 (45%); 160 (22%); 246 (33%)
Mean BMI (SD) (range))	26 (4) (16–38)
Mean Systolic BP (SD) (range))	150 (24) (96-232)
Mean Diastolic BP (SD) (range))	88 (24) (58-126)
Measured Hypertension (140/90 mmHg) (%)	346 (46.8)
Measured Hypertension Discordant pairs (140/90 mmHg)	87
Measured Hypertension (130/80 mmHg) (%)	504 (68.1)
Measured Hypertension Discordant pairs (130/80 mmHg)	70
Incident Hypertension (%) (total *n*)	77 (14%) (536)
Incident Hypertension Discordant (%)	49 (18.3%)

*BMI* body mass index, *BP* blood pressure, *MZ* monozygotic, *SD* standard deviation

### Systolic and diastolic blood pressure measurements and hypertension diagnoses

Five phenotypes were investigated: (1) systolic BP was measured twice at baseline as a standard systolic BP (mmHg) of the upper right arm measured sitting resting with a one-minute break. The first measurement was used in the study, due to missingness in the second measurement, although the two measurements showed very similar distributions (data not shown). If an individual had reported use of a drug for treatment of hypertension, a value of 15 was added to the systolic BP value. This adjustment for anti-hypertension drugs relies on the average treatment effects of the medication [26], and it is recommend by [27] and [28], who argues that no adjustment or adjustment for instance by including a binary variable for medication use or exclusion of individuals receiving anti-hypertension drugs can lead a reduced statistical power and to shrinkage in the estimated effects of the contributing factors involved in blood pressure. This simple and straightforward approach has been shown to perform well across a variety of different but realistic clinical populations, and is likewise applied in several studies, including [12] and [14]. The hypertension drugs were classified by the ATC classification guideline (ATC codes C02, C03, C07, C08, and C09) [29].

(2) Diastolic BP was measured at baseline as the standard diastolic BP (mmHg) of the upper right arm as described for systolic BP. For individuals reporting the use of a hypertension drug, a value of 10 was added to the diastolic BP value, similarly to [14] and as explained above.

(3 and 4) A hypertension phenotype was defined as the use of an antihypertensive drug, or systolic BP and diastolic BP measurements above 140 (mmHg) and 90 (mmHg), respectively. This definition was used to enable comparison to previous studies using such definition. Secondly, an additional hypertension phenotype using a cutoff of 130 (mmHg) systolic BP and 80 (mmHg) diastolic BP was also created to follow the newest definition [4].

(5) Incident hypertension phenotype was defined based on the following International Classification of Diseases (ICD) codes: ICD-8: 400-404 and ICD-10: I10-I15 [30]. The ICD codes were extracted from the DNPR containing all hospital discharges and outpatient visits from all Danish hospitals since 1977 [22], where diagnoses were available until the 23^rd^ of November 2022. DNPR contains ICD-8 and ICD-10 codes, with ICD-10 since 1994, whereas ICD-9 codes were never implemented in Danish registries. Furthermore, the ICD codes were defined according to The Danish Health Data Authority browser [31], the Statistics Denmark containing the historical information on disease grouping [32], and the definitions by the Danish Cause of Death Registry [33]. Information on survival status was retrieved from the Danish Central Person Register [34] and was used for analysis of time-to-diagnosis by Cox regression analysis (see below).

The first occurring hypertension diagnose date was identified per person, including both main and secondary diagnoses. Then, the individuals were separated into either prevalent or incident cases, i.e., whether the first occurring diagnosis date was before (prevalent) or after (incident) blood sampling. The prevalent cases were excluded in the analyses of incident hypertension (see below), as prevalent hypertension events can potentially be reflected in a blood sample taken after disease event, whereas biological variation identified in analysis of incident hypertension events in individuals with no diagnosis before blood sampling in principle are predictive of future hypertensive events. Lastly, the number of incident discordant twin pairs were identified, defined as twin pairs in which one twin obtained a hypertension diagnosis after blood sampling, while the co-twin remained disease-free or died, and twin pairs in which both twins obtained a diagnosis yet at different time points.

### Genome-wide omics data from same individuals

Detailed descriptions of the applied Infinium HumanMethylation450K BeadChip (Illumina, San Diego, CA, United States) and Agilent SurePrint G3 Human GE 8 × 60 K Microarray (Agilent Technologies) gene expression data can be found in [23, 35, 36]. Annotation of the CpGs of the DNA methylation and the probes of transcriptomic data based on GRCh37/hg19, similarly to [23].

Lastly, for both types of omics data, the biological material from both cohorts had been analyzed and quality controlled on two different occasions, hence, each dataset had been processed individually before the datasets were merged in the present study, similarly to [23]. A variable, called dataset, was considered in the statistical analyses (see below). Only reoccurring CpGs and gene expression probes across the two cohorts were used for the present study, resulting in 451,856 CpGs and 45,698 probes for statistical analyses. In the overlap between these 451,856 CpGs and 45,698 probes, there exist 19,851 unique genes. To achieve a distribution more fit for statistical testing, the β-values of the DNA methylation data were transformed to M-values by quantile transformation [37].

### Survey data considered as covariates

The following covariates were included: age, sex, self-reported smoking status (never, former, and current smoker), body mass index (BMI), as they have previously been reported to relate to the variation and/or change in blood pressure [38]. Also, as blood cell composition can bias molecular epidemiology studies performed in blood samples, hematological cell counts were also considered. They were based on the leukocyte subtypes (neutrophiles, monocytes, basophils, eosinophils, and lymphocytes), which were available for 656 (89%) out of the 740 individuals. Cell counts for the remaining individuals had been imputed using epigenome-wide data with a modified version of the PredictCellComposition method (see [39]).

Lastly, two principal component analyses (PCA) were initially conducted (similarly to [35] and [23]) of all considered survey and register data in relation to DNA methylation data and the gene expression data. The aim was to investigate the correlation between potential confounder variables and the principal components (PCs) of the omics data. Afterwards, either the confounder variable or the PC was included in the statistical analysis. In the PCA, PC2 of the gene expression data, and PC3 of the DNA methylation data, correlated with cell counts (data not shown).

### Statistical analyses

Data handling, cleaning of survey and register data, and statistical analyses were done in R version 4.1.0 with application of the tidyverse, ggpubr, parallel, and broom.mixed R libraries. The lme4 library was used to create linear regression analyses, whereas the Survival library was in the Cox regression analyses. Adjusting for multiple testing was done using the Benjamini-Hochberg FDR correction method [40].

#### Analyses depending on blood pressure

EWAS and TWAS were conducted with the use of linear regression models in which the outcome variable was either the single DNA methylation level (CpG) or gene expression level (probe), respectively, and the blood pressure value as the explanatory variable. EWAS and TWAS were done both at the individual-level and twin pair-level.

The individual-level analysis was performed using a linear mixed effect regression model where twin pair ID was used as a random factor to consider the within-pairs’ dependency. We included the following covariates: age at blood sampling, sex, BMI, and smoking status as fixed effects, and a variable called dataset as a random effect. Also, PC3 and PC2 were included for EWAS or TWAS, respectively, reflecting the cell counts.

In the twin pair level analysis, intrapair differences in twin pairs discordant for systolic BP and diastolic BP, respectively, measurements were investigated. Four twin pairs had identical systolic BP values and were excluded together with 18 twins, who had no co-twin, leaving 357 twin pairs. For diastolic BP, ten twin pairs had identical diastolic BP values, leaving 351 twin pairs. The intra-pair difference was calculated by subtracting the lowest BP value of the co-twin from that of the co-twin with the highest BP value. Similarly, the intrapair difference in DNA methylation level, or gene expression level, were calculated and used as the outcome, whereas the intra-pair BP differences were used as exposures in a linear regression model. We adjusted for smoker status and BMI and PC2 in TWAS or PC3 in EWAS. The dataset variable was not included, as the biological samples from the same twin pair had been analyzed on the same array and within the same cohort. Likewise, we did not adjust for sex and age, as sex and age were identical in the twin pairs.

Lastly, for hypertension we found 346 hypertension cases (46.7%) and 87 discordant twin pairs using the systolic BP ≥ 140 mmHg and diastolic BP ≥ 90 mmHg definitions (hereon called hypertension 140/90) and 504 hypertension cases (68.1%) and 70 discordant twin pairs using the systolic BP ≥ 130 mmHg and diastolic BP ≥ 80 mmHg definitions (hereon called hypertension 130/80).

#### Analyses of incident hypertension based on register diagnoses

For analysis of the incident hypertension cases, Cox proportional hazards regression was conducted, both at the individual-level and twin pair level. Here, we excluded the prevalent cases (*n* = 133), individuals who had emigrated (*n* = 2), and individuals who had received antihypertensive drugs (*n* = 69), leaving 536 twins for the incident analyses.

In the individual-level analysis, the individuals were analyzed regarding time to first diagnosis (incident cases), time to death, or end-of-follow-up (individuals remaining diagnosis-free). In total, 77 individuals obtained a diagnosis, 43 individuals died, and the remaining 416 individuals were diagnosis-free to end-of-follow-up. Age was used as the underlying timescale (delayed entry at blood collection) to ensure proper age adjustment and to allow a non-linear relation between the molecular marker analyzed and the risk of hypertension. We adjusted for sex, smoking status, BMI, and PC2 in TWAS or PC3 in EWAS, and within pair dependence to consider the twin structure.

In twin pair level analysis, 49 discordant twin pair were included. In three twin pairs both twins obtained a diagnosis to different time points, and in 46 pairs one twin obtained a diagnosis, while the co-twin died or remained diagnosis-free to end-of-follow-up. This analysis was performed using the stratified Cox regression, where the baseline hazard function was pair specific and with the same co-variates as above.

### Bioinformatics analyses of overlapping genes

We conducted gene set enrichment analyses (GSEA) to explore the biological pathways related to the five phenotypes in which the identified CpGs and probes take part. For each phenotype, we found the overlap in genes between the four analyses (individual-level EWAS, twin pair-level EWAS, individual-level TWAS, twin pair-level TWAS) corresponding to b in Fig. 1. For a detailed description of the approach to identify these overlapping genes see figure text in Fig. 1. This resulted in five overlaps of genes (i.e. five phenotypes) identified in the individual-level analyses and confirmed in the twin pair level analyses, i.e., the genes confirmed when reducing the potential confounding due to shared genetic factors and early-life environment. Such genes must be considered the most relevant genes when exploring the association of molecular markers to hypertension. These five overlaps were used for GSEA with application of the Kyoto Encyclopedia of Genes and Genomes [41]. Moreover, genes present in some, or all, of the five overlaps of confirmed genes were explored. Finally, Interaction Networks were also conducted for the five overlaps of confirmed genes by application of StringApp in Cytoscape [42]. In the displayed networks, genes not connected to other genes were not visualized, and for complex networks only the most connected genes are visualized. These steps were similarly done using a cut off *p* value < 0.05 instead of an FDR < 0.05 to explore the overlapping genes based on *p* value < 0.05.

## Results

In the present study, we include 740 twins to investigate the association between DNA methylation and gene expression levels together with five phenotypes: systolic BP, diastolic BP, measured hypertension defined as 140/90 mmHg (or use of antihypertensive drug), measured hypertension defined as 130/80 mmHg (or use of antihypertensive drug), and incident diagnosis of hypertension. Association analyses are performed both at the individual-level and twin pair-level in which the latter effectively reduces the potential confounding induced by genetic and early-life environment. The study design is depicted in Fig. 1, and characteristics of the study population are summarized in Table 1.

### Findings from epigenome-wide and transcriptome-wide association studies

The results of the individual EWAS and TWAS with p values below 0.05 are listed in Supplementary Results, part 1; across all 20 analyses 726–4,416 probes and 16,333-40,719 CpG sites were found, while 0-11 probes and 0-87 CpG sites passed correction for multiple testing (FDR < 0.05). These probes and CpGs were annotated to 542–3,322 and 11,436–19,422 unique genes, respectively (summarized in Supplementary Results, part 2, Table S1).

### Genes in overlap between epigenome-wide and transcriptome-wide association studies

No overlapping genes were found using a cut-off of FDR < 0.05 within the five phenotypes, thus, we explored the CpG sites and probes with p values below 0.05. The results are listed in Supplementary Results, part 2. There were found 182, 223, 24, 31, and 108 genes in the overlap between the individual-level or twin pair-level analyses for systolic BP, diastolic BP, Hypertension 140/90, Hypertension 130/80, and incident hypertension, respectively (corresponding to b in Fig. 1) (Supplementary Results, part 2, Table 1). These genes were further investigated in GSEA and interaction networks.

### Gene network and gene set enrichment analyses of genes identified in both the individual and twin pair-level analyses

All the bioinformatic analyses can be found in Supplementary Results, part 2.

**Fig. 2:**
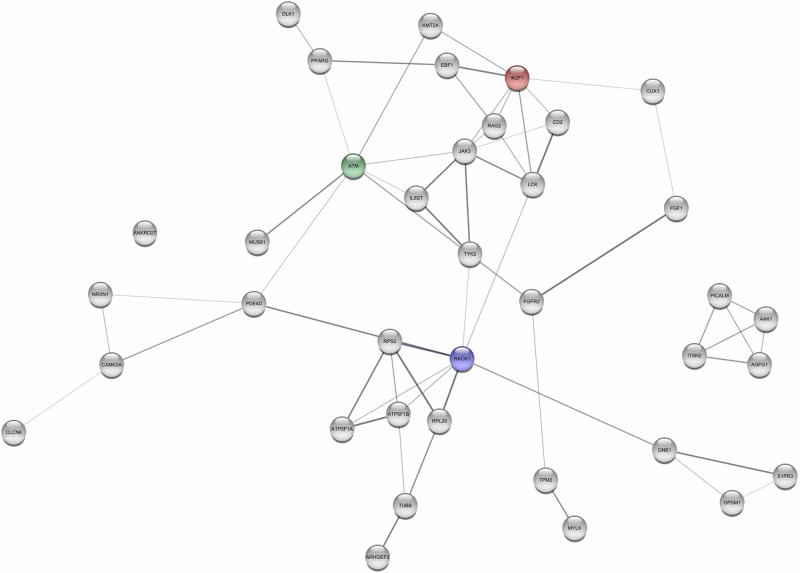
Interaction networks for Systolic Blood Pressure. Notes: green: the most connected genes, blue: the second-most connected gene, red: the third-most connected gene. Only genes having minimum 3 connections are displayed

**Fig. 3:**
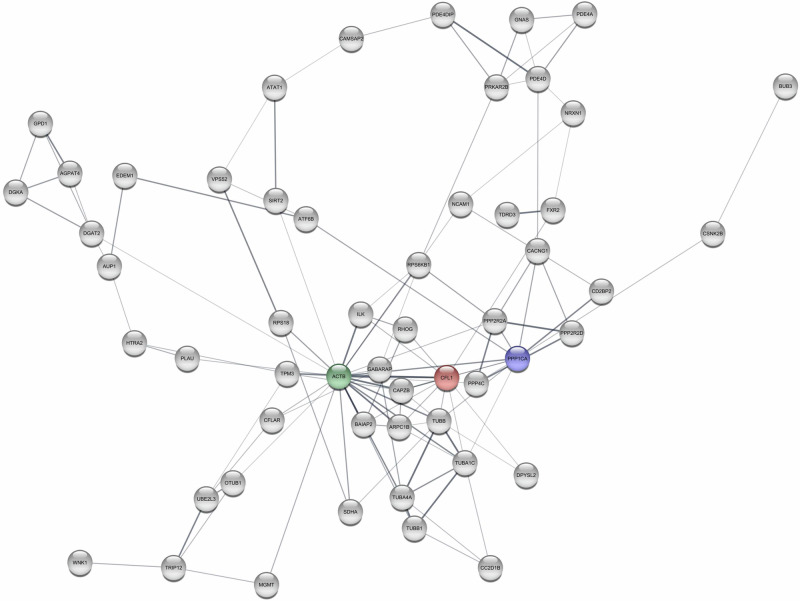
Interaction networks for Diastolic Blood Pressure. Notes: green: the most connected genes, blue: the second-most connected gene, red: the third-most connected gene. Only genes having minimum 3 connections are displayed

**Fig. 4:**
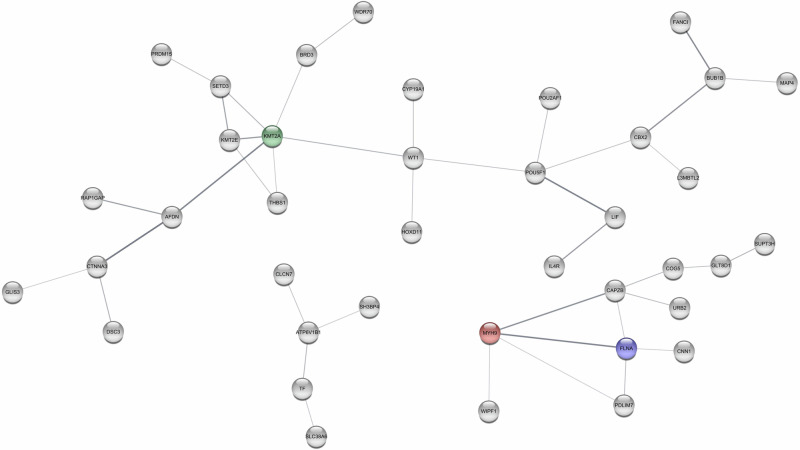
Interaction networks for Incident Hypertension. Notes: green: the most connected genes, blue: the second-most connected gene, red: the third-most connected gene. Only genes connected to networks larger than 3 are displayed

For GSEA, the 24, 31, and 108 overlapping genes found for Hypertension 140/90, Hypertension 130/80, and incident hypertension did not reveal any statistically significant pathways, while the 182 and 223 genes identified for systolic BP and diastolic BP, respectively, found two and nine pathways (see Table 2). Table 2 also shows the most connected genes in the interaction networks, while Figs. 2–4 displays their interaction networks identified for the 182 systolic BP genes, the 223 diastolic BP genes, and the 108 incident hypertension genes, respectively. The 24 genes found for Hypertension 130/80 did not display any networks, whereas the 31 genes found for Hypertension 140/90 revealed maximum one connection and is, hence, not displayed. Noteworthy, *KMT2A* (lysine methyltransferase 2 A) is the most frequently identified gene across the analyses, as it is found for the incident hypertension, systolic BP, and diastolic BP.

**Table 2:** Results of the overlapping genes

Phenotype	Genes in overlap^a^	Pathways identified by GSEA	KEGG entry	Top connected genes	
				Genes	NG
Incident Hypertension	134	0	-	KMT2A FLNA MYH9 POU5F1 WT1	6 4 4 4 4
Systolic BP	182	Calcium signaling pathway Primary immunodeficiency	hsa04020 hsa05340	ATM RACK1 IKZF1	11 10 8
Diastolic BP	223	Pathogenic Escherichia coli infection Gap junction Tight junction Fc gamma R-mediated phagocytosis Vibrio cholerae infection Glycerophospholipid metabolism Regulation of actin cytoskeleton Dilated cardiomyopathy Glycerolipid metabolism	hsa05130 hsa04540 hsa04530 hsa04666 hsa05110 map00564 hsa04810 hsa05414 map00561	ACTB PPP1CA CFL1	31 15 12
Measured Hypertension (140/90 mmHg)	31	0	-	ZBED4 TBC1D22A ESYT3 C2CD2L	1 1 1 1
Measured Hypertension (130/80 mmHg)	24	0	-	0	0

^a^Genes in overlap are genes identified between the four analyses (individual-level EWAS, twin pair-level EWAS, individual-level TWAS, twin pair-level TWAS) for a given phenotype (corresponding to b in Fig. 1). GSEA was based on KEGG Legacy gene set

*NG* Nodes degree (connections), *GSEA* gene set enrichment analyses, *KEGG* Kyoto Encyclopedia of Genes and Genomes

Gene Abbreviations: ACTB: beta-actin, ATM: ataxia-telangiectasia mutated, C2CD2L: C2CD2 like, CFL1: cofilin 1, ESYT3: extended synaptotagmin 3, FLNA: filamin A, IKZF1: IKAROS family zinc finger 1, KMT2A: lysine methyltransferase 2A, MYH9: myosin heavy chain 9, POU5F1: POU class 5 homeobox 1, PPP1CA: protein phosphatase 1 catalytic subunit alpha, RACK1: receptor for activated C kinase 1, TBC1D22A: TBC1 domain family member 22 A, WT1: WT1 transcription factor, ZBED4: zinc finger BED-type containing 4

## Discussion

Globally, hypertension is the most frequent health-related condition [2], and studies show that both epigenetic and transcriptomic factors associate to the development of hypertension [6, 12–18, 21]. Surprisingly, to the best of our knowledge, no studies have investigated both layers of biological variation in relation to hypertension, an integration which potentially gives a more holistic view compared to studies with one type of single omics data. As the association between such molecular markers and a given phenotype is biased by genetic factors, twin studies are excellent for this purpose. Hence, we here investigate epigenetic and transcriptomic data with respect to blood pressure and hypertension phenotypes in 740 twins, conditioning the findings on the less biased twin pair analysis.

In the present study, CpGs and probes with a *p* value below 0.05 were initially identified. These were annotated to their respective genes and the overlap in unique genes between the two types of molecular data and between the individual-level analyses and twin pair-level analyses wherein each phenotype was found. Such overlapping genes were investigated by GSEA and networks analyses. Interestingly, of the cross-sectional measurements at baseline only the blood pressure measurements revealed significant pathways in the GSEA and networks analyses, whereas the prevalent hypertension phenotypes did not. This could reflect the statistical advantage of investigating a numerical phenotype, as compared to a binary phenotype, or that the relevant biology more reflects a continuum than a cut off. Thus, unitary changes in BP seem to provide a better understanding of the BP regulation mechanisms and enables a finer mapping in BP differences that is likewise argued in [21]. In the literature, multi-omics studies applying continues variations in BP as phenotype are limited, yet several diseases are associated with linear or nonlinear increases in BP, such as CVD [43, 44]. This further heightens the relevance of the current multi-omics study investigating BP on a continuous scale. Moreover, systolic BP reflects how hard the heart pump blood into the arteries, whereas diastolic BP reflects the pressure on the arteries when the heart rests between beats. Studies have suggested that systolic BP is a better predictor of hypertension, although addition of diastolic BP improves risk prediction [45]. Also, many studies show that systolic blood pressure is a better predictor of risk of stroke and heart diseases [46]. Hence, it might not appear surprising that in the present study different biology is found for the two blood pressures. Finally, the analysis of incident hypertension (excluding the prevalent cases) did reveal networks in the network analysis, while the analysis of prevalent hypertension (at baseline) did not. This could indicate that the molecular markers more precisely predict future hypertension than the present variation at base line. Hence, the difference in results between the phenotypes were not unexcepted, as the models differ in type of outcome analyzed (numerical vs. binary categorical) and the temporality of the measurements (cross-sectional measurements at baseline vs. prospective incident hypertension cases). Thus, it is most likely that different biology contributed to the variation of the different phenotypes. In any case the study does report more general markers across phenotypes such as the *KMT2A* (further described below).

In the GSEA of measured blood pressure, the identified 11 pathways related to four different hierarchical pathway groups: (1) human cardiovascular disease (one pathway), (2) lipid metabolism (two pathways), (3) the immune system or human infectious diseases (four pathways) and (4) cellular processes related to signal transduction, cellular community and cell motility (four pathways). The latter could very well reflect vascular smooth muscle cell biology.

Regarding the immune system, several studies have linked its disturbances to hypertension in which especially processes related to inflammation are frequently identified [14, 15, 18, 21, 47, 48]. For instance, the FC gamma R-mediated phagocytosis pathway (hsa04666) observed here for blood pressure plays an important role in the immune system. The pathway involves phagocytosis in which the FC gamma receptors recognize foreign extracellular materials evoking intracellular signaling, and thereby, regulates several inflammatory processes [49, 50]. The pathway can also affect nitric oxide (NO) production, which is involved in vasodilation and atherosclerosis, both of which can influence blood pressure [47, 50, 51]. Also, there is epidemiologic evidence connecting processes involving the abovementioned pathway with incident hypertension [50, 52]. Lastly, components from the immune system can promote hypertension by disrupting normal renal excretory functions [48, 53]. This highlights the potential mechanism between the immune system and hypertension development.

Concerning vascular smooth muscle cells, multiple studies have linked their mechanisms to hypertension, as they play an essential role in regulation blood pressure, blood flow, microcirculation, and other cardiovascular functions [54, 55]. For instance, we identified the “Regulation of actin cytoskeleton” pathway (hsa04810), where actin cytoskeleton plays an important role in maintaining the structural integrity of smooth muscle cells [55]. A distorted vascular structure is a major cause of hypertension and cardiovascular events. Especially, an increase in vascular resistance is a key pathophysiological mechanism that promotes hypertension development, in which the increased vascular resistance is often caused by reduction in vascular diameter [47]. Moreover, a recent proteomic study showed that proteins related to actin cytoskeleton were associated with hypertension [56]. Additionally, we found the “Calcium signaling” pathway (hsa04020), which among other relates to the cytosolic calcium concentration. Alterations in this concentration are the primary mechanism for regulation of vascular smooth muscle contraction [47]. Impaired endothelial calcium signaling associates to hypertension [57]; interestingly, the calcium signaling also controls inflammatory responses which further indicate the importance of the immune system in hypertension [58, 59]. Lastly, calcium can directly pass to the cytoplasm of adjacent cells via gap junctions [60] in which the “Gap junction” pathway (hsa04540) was found. Gap junctions are vital for cell-to-cell communication, which is fundamental for proper vascular function. Gap junctions are intercellular channels that comprise connexin proteins that are associated with vascular disease development such as hypertension and atherosclerosis [60, 61].

The most connected genes (hub genes) for the interaction network were lysine methyltransferase 2A (*KMT2A)* for incident hypertension, ataxia-telangiectasia mutated (*ATM)* for systolic BP, and beta-actin (*ACTB)* for diastolic BP.

Overall, *KMT2A* plays a key role in embryonic development, neurodevelopment, and hematopoiesis, while altered *KMT2A* expression is associated with multiple pathologic conditions, especially blood cancers such as acute myeloid leukemia [62]. *KMT2A* encodes a lysine methyltransferase that is a large and complex protein, which has a fundamental role in gene expression, as it is responsible for transcription activation by methylation of lysine 4 of histone 3 (*H3K4*) [62]. *H3K4* methylation regulates gene expression through chromatin remodeling by the Nucleosome Remodeling Factor complex [63] and is for instance involved in nitric oxide (NO) formation, which also relate to *ATM* and *ACTB*, and is described later. Moreover, KMT2A is involved in a macromolecular complex that regulates the transcriptional activity of homeobox (*HOX*) genes [64]. *HOX* are a conserved family of transcription factors that is known to be involved in cardiovascular disease development [65] and have a key role in atherosclerosis development, for instance by involvement in inflammatory response and vascular remodeling, which can result in hypertension [65]. *HOX* facilitates correct development of immature hematopoietic progenitors to functional immune cells [66]. Several immune cells are reported as essential contributors to atherosclerosis and CVD development, and hematopoiesis alterations associate to common risk factors of CVD, such as hypertension [67]. However, the exact molecular mechanisms of KMT2A-HOX influencing hypertension are unclear and only a few HOX-dependent pathways have been characterized. Additionally, *HOX* also possesses non-transcriptional functions, such as roles in DNA damage repair [65]. Noteworthy, *KMT2A* was found to be an overlapping gene in the incident hypertension, systolic BP and diastolic BP analyses which further highlights the importance of *KMT2A* related to blood pressure and hypertension.

*ATM* encodes a serine/threonine kinase that plays a crucial role in DNA repair pathways, and as a sensor of oxidative stress, for which dysregulation has been reported to be connected to increased inflammation in pulmonary arterial hypertension [68]. The ATM variants are shown to worsen features of metabolic syndrome, including hypertension, and are associated to immune deficiency and CVD [23, 69, 70]. Molecularly, *ATM* can phosphorylate and active NF-κB, which impacts the later described NO production [71, 72].

*ACTB* encodes a highly conserved actin protein that is involved in cell motility, structure, integrity, and intercellular signaling and in the abovementioned “Regulation of actin cytoskeleton” pathway [73]. Furthermore, *ACTB* plays a role in the regulation of NO formation [74, 75]. Lastly, *ACTB* has also been found to be associated to CVD [23, 74, 76].

Taken all together, both *KMT2A*, *ATM*, and *ACTB* can be related to NO. Looking in the literature, evidence show that NO dysregulation is one of the earliest event in the development of hypertension [77]. NO is a signaling molecule that possesses anti-atherogenic properties and is involved in the improvement of vasorelaxation and endothelial function, regulation of blood pressure, as well as inhibition of oxidative stress and inflammation [72, 78]. NO is produced by NO synthase (NOS), of which three isoforms exist: endothelial NOS (eNOS), neural NOS, and inducible NOS (iNOS). The two formers are constitutive isoforms that produce NO in levels that have cardioprotective properties, where the cardioprotective mechanisms of NO are further described in [78], and primarily relate to dilation of the blood vessels as well as anti-inflammatory response.

Upon regulation of *H3K4* by *KMT2A*, iNOS becomes activated increasing blood pressure [78–80]. Increased *iNOS* activity results in abnormal high levels of NO, of which NO can react with superoxide anions yielding peroxynitrite inducing nitrosative stress, inflammation, and endothelial dysfunction potentially leading to hypertension [78]. Moreover, upregulated iNOS activity can promote arginase activity that enables it to compete with eNOS for L-argnine, which reduces the bioavailability of NO and uncouples eNOS but instead maintains the formation of harmful peroxynitrite [78]. Upon phosphorylation and activation of NF-κB by *ATM*, eNOS activity is reduced leading to low levels of NO inducing vasoconstriction, oxidative stress, and inflammation which can lead to hypertension [71, 72]. *ACTB* has been shown be associated with NO production by eNOS, where dysregulation of *ACTB* can result in aforementioned complications [81]. Finally, the reduced bioavailability of NO, either by inhibition of eNOS or activation of iNOS, is associated with several cardiovascular diseases and conditions, such coronary diseases, atherosclerosis, and hypertension [78].

Lastly, to the best of our knowledge none of the previous EWAS or TWAS published on hypertension related phenotypes have reported KMT2A, *ATM*, and *ACTB* among their top findings. However, this might not be surprising, as these studies did not condition their findings on both EWAS and TWAS relevance as done in the present study. Hence, the genes identified in our study (across two layers of data) might be overlooked in their findings, as they might not be among the top findings in their one type of data.

The current study has several strengths. Firstly, the use of twin data makes it possible to investigate the association between the molecular markers and the phenotypes, while reducing the effect of confounders, such as genetic and early-life environment. Moreover, use of nationwide register data for analysis of incident hypertension is an advantage, as it is less biased than longitudinal analyses of self-reported data. However, the study also has some limitations. Firstly, as no overlapping genes were found when adjusting for multiple testing, we conducted an explorative study investigating genes with *p* values below 0.05. Thus, it is possible that false positives exist among the reported genes. Therefore, replication studies are needed to verify our findings. However, previous systematic comparison of the power of the classical case-control study to the power of the discordant twin pair design, showed that the latter reduces the needed sample sized from 221 to 23 twin pairs to obtain a power >0.80 for a trait with a heritability of 60% [20]. Thus, as hypertension has been reported to have a heritability of 30–50% [8] and as we included 49–87 twin pairs, the power of the twin pair analyses appears to be reasonably good. The individual-level analysis (including 77–504 cases) is, on the other hand, most likely underpowered for the most part. In any case, as the findings of the present study are conditioned on the findings of the twin pair analyses, this is less of an issue, and it emphasizes the strength of including twin pairs in molecular studies compared to solely investigating singletons. Also, in the incident analysis, we only included individuals who did not have a diagnosis before blood sampling. However, as hypertension develops over years, we cannot exclude that pathological alterations were already present at blood sampling. Nevertheless, performing sensitivity analyses excluding cases with a time to hypertension diagnosis between 2–7 years after blood sampling led to similar results as reported here (data not shown), which indicates that this is less of an issue in the present data. Lastly, the application of blood samples implies that the measured molecular markers represent the general biology of the individuals, and not the biology of a specific part of the body, such as the endothelial cells of the blood vessels. On the other hand, the use of blood samples to identify molecular markers has great potential due to their non-invasive nature of sampling.

In conclusion, we studied blood pressure measurements and incident hypertension diagnoses from a nationwide registry for 740 twins for whom both genome-wide epigenetic and gene transcription data were available. By examining the genes identified in both omics layers, at both the individual-level and twin pair-level, we found novel candidate genes and pathways in which especially the *KMT2A* gene and pathways related to the immune system and vascular smooth muscle cells appear appealing. These markers could be advantageous for understanding blood pressure regulation, and the prevention of future hypertension. However, additional studies for verification are needed.

## Supplementary information

Supplementary Information

Supplementary Information

Supplementary Information

Supplementary Information

Supplementary Information

Supplementary Information

## Data Availability

According to Danish and EU legislation, the transfer and sharing of individual-level data requires prior approval from the Danish Data Protection Agency and requires that data sharing requests be dealt with on a case-by-case basis. Therefore, the data from the present study cannot be deposited in a public database. However, we welcome any inquiries regarding collaboration and individual requests for data sharing.
